# Neuroprotective effects of blockers for T-type calcium channels

**DOI:** 10.1186/1750-1326-4-44

**Published:** 2009-10-28

**Authors:** Norelle C Wildburger, Avary Lin-Ye, Michelle A Baird, Debin Lei, Jianxin Bao

**Affiliations:** 1 Department of Otolaryngology, Center for Aging, Washington University, 4560 Clayton Avenue, St Louis, MO 63110, USA

## Abstract

Cognitive and functional decline with age is correlated with deregulation of intracellular calcium, which can lead to neuronal death in the brain. Previous studies have found protective effects of various calcium channel blockers in pathological conditions. However, little has been done to explore possible protective effects of blockers for T-type calcium channels, which forms a family of FDA approved anti-epileptic drugs. In this study, we found that neurons showed an increase in viability after treatment with either L-type or T-type calcium channel antagonists. The family of low-voltage activated, or T-type calcium channels, comprise of three members (Ca_v_3.1, Ca_v_3.2, and Ca_v_3.3) based on their respective main pore-forming alpha subunits: α1G, α1H, and α1I. Among these three subunits, α1H is highly expressed in hippocampus and certain cortical regions. However, T-type calcium channel blockers can protect neurons derived from α1H-/- mice, suggesting that neuroprotection demonstrated by these drugs is not through the α1H subunit. In addition, blockers for T-type calcium channels were not able to confer any protection to neurons in long-term cultures, while blockers of L-type calcium channels could protect neurons. These data indicate a new function of blockers for T-type calcium channels, and also suggest different mechanisms to regulate neuronal survival by calcium signaling pathways. Thus, our findings have important implications in the development of new treatment for age-related neurodegenerative disorders.

## Background

Calcium signaling pathways play a vital role in the survival of neurons. With increasing age, calcium homeostasis can be disrupted in the brain, which leads to cognitive and functional decline [1-6]. Thus it raises the possibility of protecting neurons by identifying chemicals able to modulate calcium homeostasis in neurons during aging.

Calcium homeostasis can be regulated by several types of calcium channels, including voltage-gated calcium channels (VGCCs). VGCCs can be divided into two groups: high-voltage activated calcium channels such as L-type calcium channels and low-voltage activated calcium channels such as T-type calcium channels [7,8]. The family of T-type calcium channels comprise three members (Ca_v_3.1, Ca_v_3.2, and Ca_v_3.3) based on their respective main pore-forming alpha subunits: α1G, α1H, and α1I [9,10]. T-type calcium channels are predominantly found in neurons [11,12], but have been found in other cells including smooth muscle myocytes, pacemaker cells of the heart, glial cells, fibroblasts, osteoblasts, retinal cells, and adrenocortical cells [13-15]. L-type channels also have a wide distribution in central nervous system [16].

Blockers for both L-type and T-type calcium channels have been developed to treat various diseases. Trimethadione (TMO) is a T-type calcium channel blocker approved by the FDA as an anticonvulsant for absence seizures. Interestingly, TMO can also ameliorate noise-induced hearing loss (NIHL) by preserving the outer hair cells [17] and extend the life span of *C. elegans *[18]. Another blocker for T-type calcium channels, mibefradil, is a particularly effective inhibitor of the Ca^+2 ^influx mediated by the α1H (C_av_3.2) subunit [19]. In previous studies, it has shown to increase rat survival with chronic heart failure [20] and limit infarct size [21] with weak inotropic effects [22-24]. Mibefradil can protect neurons under oxygen-glucose deprivation events and post-ischemic conditions [25]. Blockers for L-type calcium channels such as nimodipine have been shown to increase survival after global ischemia [26], prevent apoptotic and necrotic cell death after transient focal ischemia [27,28], reduce damage resulting from brain edema [29], improve patient outcome with severe head injuries, related secondary neuronal damage [30], and subarachnoid hemorrhage [31]. However, the possible molecular mechanisms for the beneficial effects of T-type and L-type calcium channel blockers are largely unknown, mainly due to complicated *in vivo *interactions. In this study, we established cell culture models to directly test whether these drugs could preserve neurons *in vitro *in both long-term and short-term cultures.

## Results

### Neuroprotection by Nimodine

**Figure 1:**
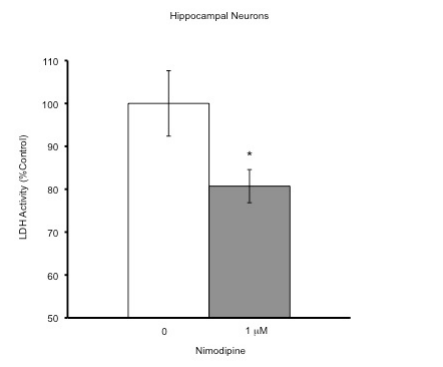
**Neuronal protection by nimodipine**. Hippocampal neurons from E18 C57BL/6J mice and cultured for 7-8 days in neurobasal medium with 2% FBS. Fresh medium was placed in wells and neurons were treated with either 0 or 1 μM nimodipine (n = 12 each). Neurons were subjected to LDH assay to quantify cell death 48 h later (10 DIV); nimodipine remained in the cultures throughout this time. Mean LDH value expressed as % of control. *p ≤ 0.05 compared with control condition.

To test whether blockers for L-type calcium channels could protect neurons in our neuronal culture model, we cultured neurons from the hippocampuses of 18 day-old neonatal (E18) C57BL/6J mice. The viability of neurons in these cultures was then analyzed using lactate dehydrogenase (LDH) assay after 8-days culture and 48 hours after treatment with nimodipine (total 10 days) at a dose of 1 μM (Fig. 1). The control was normalized to 100% and cell death was expressed as % of control. In comparison with the control there was a significant protection of hippocampal neurons by nimodipine (*t*-test, *p *= 0.027). This result demonstrated an increase in cell survival after nimodipine treatment, which suggested that the beneficial effect of the same drug in ischemia studies could be due to the direct neuronal protection [26-28].

### Neuroprotection by TMO

**Figure 2:**
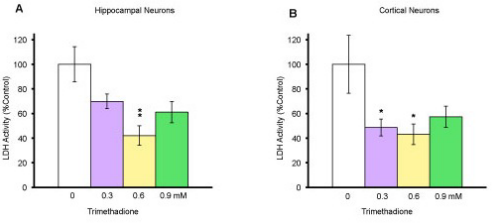
**Neuronal protection by trimethadione**. (A) E18 hippocampal neurons cultured in neurobasal medium with 2% FBS for 7-8 days. Medium was replenished at the 8^th ^day of culture and neurons treated with either 0 mM, 0.3 mM, 0.6 mM, or 0.9 mM (control, n = 16; treatment groups, n = 8 each). Cell death was performed with LDH assay 10 DIV (24 h later); mean LDH value expressed as % of control. *p ≤ 0.05 and **p ≤ 0.01 compared with the control condition. Raw data was used for one-way ANOVA. (B) E18 cortical neurons cultured in neurobasal medium with 2% FBS for 7-8 days. Medium was replenished on the 8^th ^day and neurons treated with either 0 mM, 0.3 mM, 0.6 mM, or 0.9 mM (n = 12 each). Cell viability was performed with LDH assay on 10 DIV. Mean LDH value expressed as % of control. *p ≤ 0.05 compared with the control using student's *t *test. Raw data was used for one-way ANOVA.

To test whether blockers for T-type calcium channels could protect neurons, we prepared similar neuronal cultures and treated them for 48 hours with TMO at a range of concentrations (0 mM, 0.3 mM, 0.6 mM, and 0.9 mM) in order to establish a dose curve (Fig. 2). When the cell viability was quantified in the hippocampal culture (total 10 days) with one-way ANOVA, there was a statistical significance between the drug groups and the control (*p *= 0.0090). In the hippocampal culture, no significant difference was observed between the control and groups treated with TMO at either 0.3 mM or 0.9 mM (*p *= 0.14 and *p *= 0.084 respectively). However, the group treated with TMO at 0.6 mM showed a significant preservation of hippocampal neurons (*p *= 0.008). When one-way ANOVA was performed on the cortical culture there was a statistical significance between the drug groups and the control (*p *= 0.0219). The cortical culture also showed the greatest significant preservation of neurons at the 0.6 mM dose (*p *= 0.033), and TMO at 0.3 mM also preserved cortical neurons (*p *= 0.048). These data provide first evidence for the TMO protection of both hippocampal and cortical neurons.

### Neuroprotection by Mibefradil

**Figure 3:**
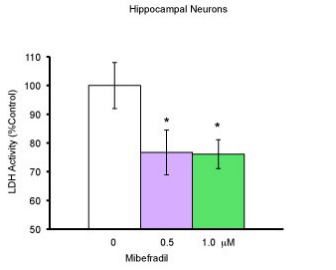
**Neuronal protection by Mibefradil**. Using cells from the hippocampi of E18 mice, neurons were cultured for 7-8 days with neurobasal medium with 2% FBS. On the 8^th ^day of culture, medium was replaced and cells treated with mibefradil in concentrations of 0 μM, 0.5 μM, and 1 μM (all groups, n = 12). Cell death was quantified using LDH assay 48 hours after treatment. Raw data was used for one-way ANOVA. When the data was expressed as % of control. *p ≤ 0.05 compared to the control was significant using student's *t *test.

To ensure that this neuroprotective effect was not exclusive to trimethadione alone, but to T-type calcium channel blockers in general, we tested similar neuronal cultures with mibefradil (Fig. 3). Using cells from the hippocampuses of E18 mice, neurons were cultured for eight days and treated with mibefradil in doses of 0 μM, 0.5 μM, 1 μM, and 10 μM. LDH was performed 48 hours after treatment. The protection was statistically significant after the treatment of mibefradil at either 0.5 μM or 1 μM (*p *= 0.0355, one-way ANOVA). The treatment of mibefradil at 10 μM was toxic to neurons (data not shown). When the data was normalized and expressed as % of control, and student's *t *test performed for each dose, mibefradil at 0.5 μM or 1 μM was significant in preserving neurons (*p *= 0.0494 and *p *= 0.019 respectively). Therefore, blockers for T-type calcium channels could protect neurons directly at the cellular level.

### The α1H subunit is not the molecular target for neuroprotection by blockers for T-type calcium channels

**Figure 4:**
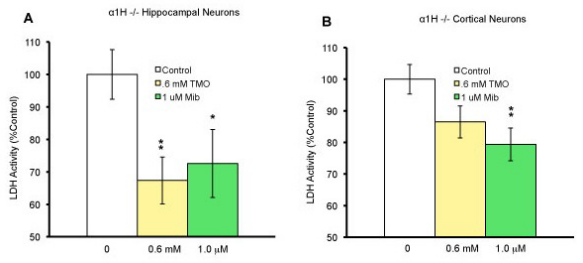
**Protection of α1H-/- hippocampal and cortical neurons by either trimethadione or mibefradil**. Neurons of E18 knockout mice missing the α1H subunit of the T-type calcium channel were cultured for 7-8 days. (A) On day 8 of culture, the medium was refreshed and the hippocampal neurons given either 0 mM, 0.6 mM TMO, or 1 μM mibefradil (control, n = 24; treatment groups, n = 12 each). After 48 hours, cell death was quantified using an LDH assay. The raw data was used to perform one-way ANOVA. Afterwards the mean LDH values were expressed as % of control. *p ≤ 0.05 and **p ≤ 0.01 compared to the control was significant. (B) Cortical neurons were given either 0 mM, 0.6 mM TMO, or 1 μM mibefradil (control, n = 24; treatment groups, n = 12 each). After 48 hours, cell death was quantified using an LDH assay. The raw data was used to perform one-way ANOVA. Afterwards the mean LDH values were expressed as % of control. *p ≤ 0.05 and **p ≤ 0.01 compared to the control was significant using student's *t *test.

Because the α1H subunit is highly expressed in the hippocampus and regions of the cortex, we tested whether this subunit was the key molecule for neuroprotection by blockers of T-type calcium channels. The hippocampal and cortical neurons derived from E18 α1H-/- mice were cultured and treated with either 0.6 mM TMO or 1 μM mibefradil. LDH assay was performed on both cultures 48 hrs after treatment (Fig. 4). Using one-way ANOVA, we found a statistical significance between the control and drug groups (*p *= 0.0152 for hippocampal neurons and *p *= 0.0106 for cortical neurons). When the data was normalized and expressed as % of control, student's *t *test was performed for each concentration. Both TMO and mibefradil could significantly protect hippocampal neurons (*p *= 0.01 and *p *= 0.044 respectively). For the cortical neurons, mibefradil demonstrated significant protective effects (*p *= 0.01). While TMO demonstrated protective effects, but they were not statistically significant (P = 0.081). These results suggested that the neuroprotective effects of both drugs were not through the α1H subunit.

### Neuroprotection in long-term cultures

**Figure 5:**
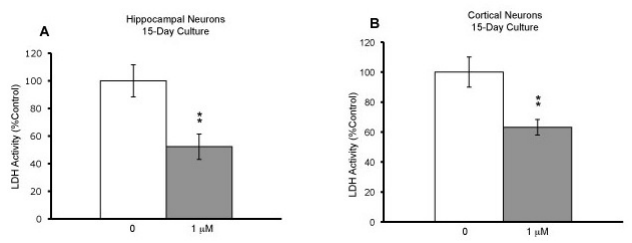
**Neuroprotection by Nimodipine in long-term culture**. Hippocampal neurons from E18 C57BL/6J mice were cultured for 7-8 days in neurobasal medium containing 2% FBS. (A) On the 8 DIV, the medium was replenished and the neurons treated with either 0 or 1 μM nimodipine (control, n = 6; nimodipine, n = 6). Cell death was measured using LDH assay on 15 DIV. Mean values were expressed as % of control ± SEM. *p ≤ 0.05 and **p ≤ 0.01 compared with the control condition. (B) Cortical neurons were treated with either 0 or 1 μM nimodipine (control, n = 6; nimodipine, n = 6). Cell death was measured using LDH assay on 15 DIV. Mean values were expressed as % of control. *p ≤ 0.05 and **p ≤ 0.01 compared with the control condition using student's *t *test.

**Figure 6:**
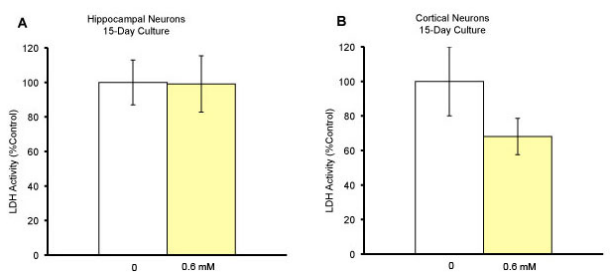
**No neuroprotection by Trimethadione in long-term culture**. (A) Hippocampal neurons were cultured for 7-8 days and treated with 0.6 mM TMO. On 15 DIV, cell death was quantified with the LDH assay. The mean values were expressed as % of control (control, n = 12; trimethadione, n = 6) (B) *In vitro *cortical neurons were cultured for 7-8 days and treated with 0.6 mM TMO (control, n = 12; trimethadione, n = 6). On 15 DIV, cell death was quantified using LDH assay. The mean values were expressed as % of control ± SEM.

We also tested whether blockers of L-type and T-type calcium channels could protect neurons in long-term neuronal culture, an "age in the dish" model [32]. Hippocampal and cortical neurons were cultured for a total of 15 days. At eight days in culture, neurons were treated with either nimodipine at 1 μM or TMO at 0.6 mM. Seven days after the treatment, LDH assay was performed. Nimodipine protected both hippocampal (*p *= 0.009) and cortical (*p *= 0.008) neurons (Fig. 5), while TMO was ineffective in protecting neurons in long-term cultures (Fig. 6; *t*-test).

## Discussion

Intracellular calcium increase is an early event triggering neuronal death in age-related neurodegenerative disorders such as Alzheimer's disease [1-6]. The L-type voltage-gated calcium channels have been implicated in playing a role in neuronal death during aging [33,34]. Though T-type calcium current increases with age [35], to our knowledge the role of low-voltage gated calcium channels in the neuronal survival has never been studied. Using a well characterized *in vitro *neuronal culture model, we first show that both blockers of L-type and T-type calcium channels can protect neurons in the dish. Although the α1H subunit is highly expressed in the hippocampus and in regions of the cortex, blockers for T-type calcium channels continued to protect neurons derived from the α1H-/- mice, indicating that the neuroprotection demonstrated by these drugs is not through the α1H subunit. Interestingly, nimodipine, a blocker for L-type calcium channels, can protect neurons in the long-term culture model, while blockers for T-type calcium channels are unable to protect neurons in the same culture model.

It has been suggested that T-type calcium channels contribute to intracellular calcium increase and cell death for both glial and neuronal cells under ischemia conditions [36,25]. Here, we show that trimethadione and mibefradil provide a very significant protection against neuronal death in the dish. Both drugs are selective inhibitors for T-type calcium channels. The neuroprotective effects of anticonvulsants on organotypic hippocampal cultures subjected to transient ischemia have been reported [37]. One possible explanation for the lack of neuroprotection with 0.9 mM trimethadione (Fig. 2) is an excessive block of calcium channel currents causing a detrimental lowering of intracellular calcium concentration in the cell. Since calcium ions play important roles as second messengers, vesicle fusion and neurotransmitter release, and axon growth cones, any drastic inhibition of Ca^+2 ^currents would also be lethal to neurons [38-44]. It is interesting that ethosuximide, phenobarbital, and phenytoin, reported to be the most neuroprotective anticonvulsants, are also the drugs with most potent T-type calcium current inhibitory activity [45,46], which suggests that these drugs protect neurons through blocking T-type calcium channels.

In addition, our results show that similar drugs can still protect neurons derived from mice lacking the α1H subunit. The neuroprotection demonstrated by T-type calcium channel antagonists in this case can be due to the presence of the other two subunits (α1G or α1I) in the cultured neurons. Therefore, it would be interesting in the future to test their protective effects in neurons derived from mice lacking either of the other two α1 subunits. In addition, blocking of other ion channels by these two drugs may also be involved in protection of neurons because both of these drugs can bind to other channels with low affinities. For example, mibefradil can block delayed rectifier potassium channels and sodium channels [47,48].

Presently, there are no effective medications for age-related neurodegeneration. Human population studies have correlated female patients taking calcium channel blocking medication with a better hearing threshold during aging [49], which suggests that altered calcium regulation might contribute to age-related loss auditory neurons. The "calcium hypothesis of neuronal aging" [5] has been supported by extensive studies, especially the role of excess calcium influx via L-type voltage-gated calcium channels and age-related changes in calcium intracellular buffering [50,1]. However, few studies have explored the role of T-type voltage-gated calcium channels in age-related neuronal loss. Therefore, we have tested whether trimethadione could protect neurons in the long-term culture. Surprisingly, no protective effects are observed by trimethadione while a significant protection is observed after blocking L-type calcium channels. Our observation raises the possibility that the survival of neurons depends not only on the level of intracellular calcium but also the source of intracellular calcium. These intracellular sources of calcium include and are not limited to the endoplasmic reticulum via the ryanodine receptors and inositole 1,4,5-triphosphate receptors [51,52]. In addition, due to the fact that T-type calcium channel blockers can increase *C. elegans *lifespan, our finding does not exclude the possibility that trimethadione may protect neurons *in vivo *through its systemic effects [18].

Currently, mechanisms for neuroprotection by these antiepileptic drugs are unknown. Although there are many differences between *in vitro *and *in vivo *conditions, culture models can be useful to dissect molecular pathways underlying age-dependent changes in neurons [53]. Our data from the neuronal cultures suggest that this culture model can be an effective model system to dissect possible protective mechanisms for these drugs. Our findings also encourage potential clinical studies to examine adults taking calcium channel blockers for their cognitive functions with age, including the risk of neurodegenerative disorders.

## Conclusion

Our findings suggest that cortical and hippocampal neurons can be protected *in vitro *by blockers for L-type or T-type calcium channels. The neuroprotection of blockers for T-type calcium channels is not through the α1H subunit. Furthermore, neurons in the long-term culture can be protected only by blocking L-type calcium channels, which suggests different molecular mechanisms for short-term and long-term survival of neurons. Our data provide insights into possible new uses of this family of antiepileptic drugs in protecting neuronal death under pathological conditions.

## Methods

### Tissue preparation

C57BL/6J and α1H-/- pregnant mice were sacrificed and the fetuses removed at E18. The pups were placed onto a Petri dish with Minimal Essential Medium plus glutamate (MEM) (Gibco, Grand Island, NY). The placenta removed and the pups placed in another Petri dish sterilized with ethanol. The E18 pups were decapitated and the brains removed without extracting the cerebellum and placed in a separate dish with MEM. The brains were sliced in half and the left and right hippocampus removed in addition to the cortex. Hippocampal and cortical sections were each transferred into separate 50 ml polypropylene centrifuge tubes- gamma sterilized (Biolgix, Shawnee Mission, KS) with the MEM using a milliliter pipette. Next, the entire medium was removed from the tube (leaving the cells at the bottom); 5 ml of neurobasal with 2% FBS (Gibco, Grand Island, N.Y.) replaced the old medium. The contents of the tube were mixed using a syringe ten times. Then, using the syringe, the contents were transferred into a 15 ml polypropylene centrifuge tube using a 40 μM nylon filter (BD Falcon, Bedford, MA). The cells were centrifuged at 1,000 rpms for 10 min When finished the medium was removed with a pipette and 5 ml of fresh neurobasal with 2% FBS added. A milliliter pipette was used to mix the contents of the tube five times and then centrifuge (1,000 rpms; 10 min). Once again the medium was removed and replaced with fresh neurobasal (no FBS; 5 ml). 11 ml of neurobasal (no FBS) was placed in a large centrifuge tube and the contents of the small centrifuge tube (total 5 ml) were transferred in to the large tube. Using a micropipette, 150 μl of the cell solution was placed into each well of a 96 tissue culture plate (Zellkultur, Trasadingen, Switzerland). The culture plates were kept in a 37°C incubator, CO_2 _at 5% (Fisher Scientific).

### Preparation of a 96 well culture plate

Culture plates were removed from their wrapping and each well was coated with 50 μl of Poly-D lysine hydrobromide (Sigma, St. Louis, MO). The plates were left to sit for 90 min with Poly-D. Each well was washed out three times using 200 μl of dH_2_0. The plates were sealed with parafilm (Ameican National Can, Greenwich, CT) and refrigerated at 4°C for later use.

### Neurobasal medium with 2% FBS

1 ml of B27 supplement (Gibco, Grand Island, NY), 125 μl of L-gluton (200 milli moles) (Sigma, St. Louis MO), 50 μl penicillin (WUSM, St. Louis, MO) 48 ml of neurobasal medium (Gibco, Grand Island, NY), and 1 ml of FBS (100%).

### Minimal Essential Medium (MEM)

1 ml of B27 supplement, 125 μl of L-gluton (200 milli moles), 50 μl penicillin, and 49 ml of neurobasal medium.

### Monitoring of cell death

To measure cell cytotoxicity, lactate dehydrogenase (LDH) assay was performed. LDH, an enzyme normally found in the cytosol of cells, is released upon damage or death to the cell. 25 μl of medium from the hippocampal or cortical cultures were placed in a 96 well tissue plate with 125 μl LDH buffer (4.53 g KH_2_PO_4 _and 11.61 g K_2_HPO_4 _in 1 liter, pH 7.4) and 100 μl NADH (Sigma, St. Louis, MO) solution (0.03% NADH in LDH buffer, freshly made) for 10 min. 25 μl of pyruvate solution (0.25% pyruvate in LDH buffer) was added right before the reading using Thermo max microplate reader (Molecular Devices, Sunnyvale, CA), with Soft Max Pro at 340 nm.

### Statistical Analysis

Results were expressed as mean ± standard error (SEM). Student's *t *test and one-way ANOVA test were used in analyzing data for LDH measurements. A p-value less than .05 was regarded as statistically significant.

## Competing interests

The authors declare that they have no competing interests.

## Authors' contributions

NCW, AL, MB, DL, and JB designed the experiments, statistical analysis, interpreted the results and drafted the manuscript. NCW and DL carried out the LDH assay and neuronal culturing. NCW and JB drafted the manuscript. All authors read and approved the final manuscript.
